# The power of support from companion animals for people living with mental health problems: a systematic review and narrative synthesis of the evidence

**DOI:** 10.1186/s12888-018-1613-2

**Published:** 2018-02-05

**Authors:** Helen Louise Brooks, Kelly Rushton, Karina Lovell, Penny Bee, Lauren Walker, Laura Grant, Anne Rogers

**Affiliations:** 1 Psychology of Healthcare Research Group, Department of Psychological Sciences, Institute of Psychology, Health and Society, University of Liverpool, Liverpool, UK; 2 Mental Health Research Group, Division of Nursing, Midwifery and Social Work, Faculty of Biology, Medicine and Health, School of Health Sciences, Manchester Academic Health Science Centre, University of Manchester, Manchester, UK; 3 University of Manchester, Manchester, UK; 4 NIHR CLAHRC Wessex, Faculty of Health Sciences, University of Southampton, Southampton, UK

**Keywords:** Pets, Mental health, Systematic review, Narrative synthesis, Self-management, Personal communities, Networks of support

## Abstract

**Background:**

There is increasing recognition of the therapeutic function pets can play in relation to mental health. However, there has been no systematic review of the evidence related to the comprehensive role of companion animals and how pets might contribute to the work associated with managing a long-term mental health condition. The aim of this study was to explore the extent, nature and quality of the evidence implicating the role and utility of pet ownership for people living with a mental health condition.

**Methods:**

A systematic search for studies exploring the role of companion animals in the management of mental health conditions was undertaken by searching 9 databases and undertaking a scoping review of grey literature from the earliest record until March 2017. To be eligible for inclusion, studies had to be published in English and report on primary data related to the relationship between domestic animal ownership and the management of diagnosable mental health conditions. Synthesis of qualitative and quantitative data was undertaken in parallel using a narrative synthesis informed by an illness work theoretical framework.

**Results:**

A total of 17 studies were included in the review. Quantitative evidence relating to the benefits of pet ownership was mixed with included studies demonstrating positive, negative and neutral impacts of pet ownership. Qualitative studies illuminated the intensiveness of connectivity people with companion animals reported, and the multi-faceted ways in which pets contributed to the work associated with managing a mental health condition, particularly in times of crisis. The negative aspects of pet ownership were also highlighted, including the practical and emotional burden of pet ownership and the psychological impact that losing a pet has.

**Conclusion:**

This review suggests that pets provide benefits to those with mental health conditions. Further research is required to test the nature and extent of this relationship, incorporating outcomes that cover the range of roles and types of support pets confer in relation to mental health and the means by which these can be incorporated into the mainstay of support for people experiencing a mental health problem.

**Electronic supplementary material:**

The online version of this article (10.1186/s12888-018-1613-2) contains supplementary material, which is available to authorized users.

## Background

The rise in people experiencing a mental health problem [1] and the identification of mental illness as the leading cause of disability adjusted life years globally (DALYs) [2, 3] requires concerted effort in identifying and mobilising resources to support people living with a mental health problem. Traditional approaches to the self-management of long-term conditions focus on psychological mechanisms of behaviour change, which have been shown to have some utility for managing symptoms. However, these approaches often fail to take into account the wider resources including material and social relationships in people’s domestic and local environments which form the latent and constituent part of systems of lay and community support [4]. These are increasingly being recognised as holding significant relevance for the management of long-term health conditions [5]. Indication of the potential benefit that pets convey to the experience of mental health comes from evidence detailing the benefits of pet ownership in relation to stress reduction, improved quality of life, and pets as promoters of social and community interaction [6–8]. Recent work has shed light on the relevance of pets in the social networks of people who have received a diagnosis of a severe and enduring mental health illness (e.g. Schizophrenia and Bipolar disorder) [9] suggesting that pets can be considered alongside other human relationships. However, the evidence base for the benefit of pet ownership for those with diagnosable mental health conditions is fragmented and unclear.

The enduring relationship between humans and domestic animals is well documented and there are an estimated 10 million cats (23% of households with one or more cat) and 11.5 million dogs (30% of households with one or more dog) kept as pets in the UK [10], with similar rates of ownership found across Europe, Australia, China and Japan [11]. Despite this phenomenon, the potential benefits that owning a pet might confer specifically to mental health has received relatively little attention. Research has focused on formalised animal contact in closed settings such as Animal Assisted Therapy (AAT). Multiple reviews have considered AAT in a variety of fields including intellectual disability [12], autism [13], general healthcare [14, 15] and neuro-rehabilitation [16, 17], but there are no systematic syntheses of the role and effects of the less structured animal contact provided by pet ownership in open settings for people with mental health conditions. The provision of ongoing support in normalised everyday settings remains an aspiration of mental health policy but the mapping of the nature of resources available and how they are, and can be, deployed remains underexplored.

### Underlying theoretical framework

This review draws on a framework of long-term condition ‘work’ informed by Corbin and Strauss [18] which was developed in the context of exploring the contribution and division of labour provided by intimate and weak ties in personal communities in relation to living with a long term condition [4, 5, 19, 20]. This approach allows for an in-depth analysis of the role of pets in relation to the tasks that need to be done to manage mental health in the context of people’s everyday lives to consolidate the evidence base in this regard. Practical work consists of tasks undertaken by network members which are practical in orientation and includes general practical activities such as housekeeping, personal care and diet and exercise activities but also illness specific practical tasks such as taking medication, understanding symptoms, making appointments and preventative work to avoid crises. Emotional work relates to wellbeing, providing companionship and being a source of comfort when worried about everyday matters or specific illness matters. Biographical work relates to the tasks and generation of ontological security, required to retain a positive sense of identity and give life meaning again post diagnosis. This involves assessments of personal expectations, capabilities, relationships and biographical events. These types of work are distributed amongst weak as well as close ties [21]. This framework has been used in preference to more traditional notions of social support as it allows for the inclusion of an in-depth understanding of the open system resources, networks and relationships that people draw on when managing a long-term condition in their everyday lives [4]. The framework was used to guide the narrative synthesis of the studies included in the review.

This review aimed to explore the nature, extent and quality of the evidence demonstrating the role of pet ownership for people with mental health conditions.

### Review questions

1. What is the nature, extent and quality of the evidence demonstrating the role of pet ownership for those with mental health conditions, with or without comorbid physical health conditions?
2. What is currently known about the mechanisms underlying any impact?

## Methods

A comprehensive search of 9 electronic databases was undertaken in March 2017. The methods and reporting of the results of this systematic review are described according to PRISMA (Preferred Reporting Items for Systematic Reviews and Meta-Analyses) guidelines [22].

### Eligibility criteria

The review sought to identify studies that reported primary data, which investigated the relationship between pet ownership and diagnosable mental health conditions. Inclusion/exclusion criteria can be found in Table 1. All participants in the sample had a diagnosable mental health condition or mental health problems associated with a diagnosed physical health condition. Papers were excluded if it was unclear who the sample were and could only be included if specific reference to diagnosable conditions was made.

**Table 1:** Inclusion/Exclusion criteria

Inclusion criteria	Exclusion criteria
English language paper	Not an English language paper
Primary data	Not primary data (e.g. systematic or review article/opinion piece)
Peer reviewed journal article/conference paper/research dissertation	Not a peer reviewed journal article (e.g. books/book chapters)
Related to pet ownership and domestic animals	Studies unrelated to pet ownership (e.g. Animal assisted therapy which does not involve the direct ownership of domestic animals)
Related to the impact of pet ownership on diagnosed mental health conditions or co-morbid mental health related to long-term physical conditions.	Not related to the impact of pet ownership on diagnosed mental health conditions or mental health components of long-term physical conditions or the nature of the sample was unclear.

Studies were not excluded by date of publication or sample size. However, those that were not published in the English language, were only published in abstract form, or were not accessible via inter-library loan were not included in this review.

### Search strategy and data sources

Electronic database searches were undertaken in March 2017 from the earliest record to March 2017 using ASSIA, CINAHL Plus, Embase, International Bibliography of the Social Sciences, Medline, PsychInfo, Social Science Full Text, Sociological Abstracts, and Web of Science. Grey literature sites were also searched including OpenGrey, Index to Theses, Electronic Theses Online Services, The Health and Social Care Information Centre Website and the Association of Health Observatories Website.

The search strategy was organised around four key areas: 1) Participants’ perspectives, 2) Pet ownership, 3) Diagnosed mental health conditions or co-morbid mental health related to long-term physical conditions and 4) impact of pets on mental health management. The search strategy was informed by published reviews, discussion within the wider project team, consideration of MeSH terms and the wider literature in the area of pet ownership. HB piloted search terms in a number of databases with input from an information technology specialist. Papers identified through piloting were assessed for additional terms, subject headings and key words with the aim of further refining the search strategy. A copy of the final search strategy is available from the author. Within each PICO component agreed search terms were combined using the Boolean operator ‘OR’ and across components using ‘AND’. The search was adapted for the individual databases and websites as required.

### Review strategy

Search results were uploaded to Endnote before removing duplicates and exporting into the data management software Covidence (https://www.Covidence.org). The first stage of the review process involved single screening at the level of title and abstract (see Table 1 for a list of inclusion and exclusion criteria). An additional reviewer independently reviewed all excluded references for validity purposes. Full texts of included articles were obtained for the purposes of full text screening. Full texts were screened for inclusion independently by two reviewers and inclusion/exclusion conflicts were resolved by a third reviewer. Acceptable concordance was predefined at 90% [23]. A concordance rate of 93% was achieved at first rating (29 exclusion/inclusion conflicts).

The reference lists of included papers were also manually searched for relevant papers. A Google Scholar alert was created in February 2017 and stopped in August 2017, which did not identify any additional articles for inclusion.

### Data extraction

Electronic forms were created in Microsoft Excel for the purpose of data extraction. Data was double extracted independently by two authors who each extracted all studies. Disagreement between extractors, which consisted of mostly minor additional detail, was resolved by consensus between authors.

The aim of the review was to explore the impact of pet ownership on diagnosed mental health conditions (or co-morbid mental health symptoms associated with other long-term conditions). Where data was available from quantitative and qualitative outcomes of mental health, these were extracted along with data relating to study design, included participants and other contextual factors.

### Quality assessment

Included articles were assessed for relevance by HB, KR and AR and for quality by HB and KR using criteria adapted from the Qualitative Research Review Guidelines - RATS and the Quantitative Assessment tool for Quantitative Studies [24]. Any disagreements were resolved by discussion between authors. The quality assessment included assessment of potential bias in terms of selection and response and the reliability and validity of the methodology utilised. No study was excluded on quality alone [25]. Studies were given one point for each quality criteria the study met (see Additional files 1 and 2) and this was used to guide the narrative synthesis of the studies included in the review.

The quality assessment process generated an average quality rating of 5.5 out of 10 for qualitative studies and 8.75 out of 10 for quantitative work (refer to Additional files 1 and 2). There were no RCTs evaluating the impact of pet ownership on diagnosed mental health conditions.

### Data synthesis

A deductive, thematic synthesis approach was constructed collaboratively between two authors (HB, KR) and the resultant analytical framework was elaborated and checked by a third (AR). Quantitative and qualitative data were synthesised and combined in parallel. Primary findings in each study were coded in line with the concepts of the networked work of illness management identified above which identified a set of three core types of work deployed by social network members of an individual’s personal community of support (*practical, emotional and biographical work*). We utilised a constant comparative approach to analysis to enhance the likelihood that concepts were translated successfully from one study to another [26]. Descriptive themes emerged which were used to describe groups of codes within each category of work. Using the framework we were able to draw comparisons between these themes and move beyond the primary findings presented within each individual paper. Individual benefits and disadvantages of pet ownership were considered in terms of the conditions and contexts they emerged from.

## Results

**Fig. 1:**
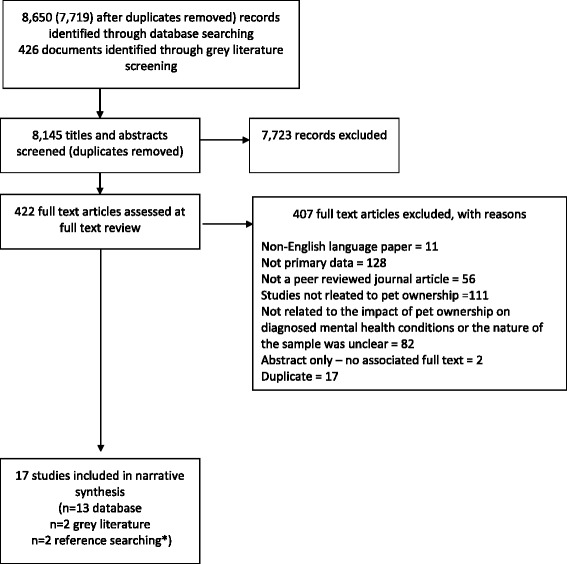
PRISMA flowchart. *Two articles identified through searching of reference lists of included articles so not included in earlier stages of the review

The search resulted in 17 studies for synthesis; the flow of studies is outlined in Fig. 1. All study characteristics and quality indicators are detailed in Additional files 1, 2, 3 and 4. Of the 17 studies, 8 were conducted in the USA [27–34], 4 in the UK [9, 35–37], 2 in Canada [38, 39] and 1 each in the Netherlands [40], Australia [41], and Sweden [42]. Twelve of the studies were reported in journal articles [9, 27, 30, 32–35, 38–42] and 5 were part of doctoral research [28, 29, 31, 36, 37]. Eight of the studies used qualitative methodology [9, 27, 28, 31, 34, 36, 37, 42], 6 were quantitative [29, 30, 33, 38–40] and 3 used mixed methods [32, 35, 41]. Methods used in the qualitative work included grounded theory [32, 36] thematic analysis [41, 42] phenomenology [28, 31] and framework analysis [9]. Quantitative studies employed cross-sectional survey design and used a variety of descriptive statistics [29, 30, 32, 33, 35, 39–41] correlational analysis [41] and regression analysis [29, 33, 35, 40].

All participants in the studies resided within the community and had either a diagnosed serious mental health condition [9, 30–32, 38, 39], mental health problems associated with a physical health condition [29, 33–35, 40, 41], mental health problems associated with a developmental disorder [37, 42] or self-reported mental health conditions [27, 28]. Two of the studies involved interviews with parents of children who had a family pet [37, 42], the remaining studies collected data directly from participants with a companion animal. Twelve of the studies included all types of companion animals [9, 27, 31–36, 39–42] and four specifically focused on either dogs and/or cats [28–30, 38]. A total of 1727 pet owners were involved in the included studies.

Of the 17 included studies, 15 reported positive aspects of pet ownership for people experiencing mental health problems [9, 27–30, 32–40, 42] and 9 reported negative elements [9, 27, 32, 35, 36, 38, 39, 41, 42]. Neutral effects of pet ownership were reported in some of the included quantitative studies, where no difference in mental health outcomes, social contacts or loneliness were reported for pet owners compared to non-pet owners [29, 32, 35, 40]. Benefits were mostly demonstrated through qualitative data, and negative elements, which were highlighted, were largely over-shadowed by co-occurring positive impact of pets in these studies [9, 27, 32, 35, 36, 38, 42].

### Themes

#### Emotional work - alleviating worry, providing comfort and mitigating against feelings of isolation and loneliness

Evidence from quantitative studies relating to contribution of pets to emotional work was mixed. There were significant findings for the benefits of canine companionship for military veterans with post-traumatic stress disorder (PTSD), including effects on reducing feelings of loneliness, depression, worry and irritability, and increased feelings of calmness [30] and there was some evidence for the direct effect of pets on depression and mood [30, 35] through close proximate contact and stroking [35]. However, this finding was not wholly supported by other quantitative studies, which reported neutral or small negative effects of pet ownership [29, 35, 40, 41]. A study investigating the effect of pet ownership and strength of attachment on depression found that pet owners were just as likely as non-pet owners to be depressed [33]. However this focussed on the sequela of depression not its alleviation or contribution of pets to managing post diagnosis. Interestingly, a study by Siegel found that pets had an effect in mediating the relationship between AIDS diagnosis and depression and that there was a weak trend towards dogs being more successful in this role than cats [33].

The importance of pets in relation to the provision of emotional work was a recurrent theme in the numerous qualitative studies included in the review where people reported a profound connection with their pet [9, 27, 30–32, 38, 39] sometimes preferring relationships with pets over relationships with other humans [37] and viewing pets as replacement family members [32]. The mechanisms through which pets made the perceived contribution to emotional work seemed to be the provision of a consistent source of comfort and affection [9, 34, 36, 41, 42]. This constant presence meant that this provision was available instantaneously without request [9, 27, 36, 42]. Pets provided calming support and were perceived to have a ‘sense’ of when it was needed.

*“The dog approaches Karin when she’s crying and comforts her by lying next to her and licking away her tears. The dog hears her, and wherever he is in the house, he comes to her. We can’t always comfort her. Sometimes Karin has said, ‘It’s a good thing we have the dog, otherwise no one would be able to comfort me”* [42].

Pets were able to provide unique emotional support as a result of their ability to respond to their owners in an intuitive way, especially in times of crisis and periods of active symptoms [9, 30, 31, 35, 36]. A related impact on loneliness was achieved through physical contact which reduced feelings of isolation [28, 36], providing a source of physical warmth and companionship [35], and by providing opportunities for communication [34, 36].

*“It is very important of people not to feel alone and isolated, and pets help you feel like you’re like everyone else. Not less than other people. My birds are very important to me and I think other people with other pets feel that way, too”* [27].

The study by Ford found that people were able to confide in their pets when they were unable to open up to other people.

*“Sometimes if I talk to the cat, perhaps it's like being in a confessional, I find I can address things that perhaps I wouldn't have done normally if I hadn't have had the cat to talk to”* [36].

In this respect, pets provided a safe environment where people can talk without fear of being judged or being a burden to others [9]. This was echoed in work where people reported that their dogs allowed them to express their feelings and clarify their thoughts without the concern that they will interrupt, offer criticism or advice, or betray confidence [30, 31].

*“They don’t have input, “this is what you should do, or maybe you should try this” or all the other commentary I get from people, who are trying to be helpful in their own right…their dying devotion and love, it’s true friendship”* [31].

The sentiment of pets being non-judgemental underpins the absence of conditionality, which was a recurring finding in included studies. Pets provided unconditional love and affection [9, 30, 31, 34, 42] which fostered self-acceptance and congruence [28]. Pets constituted a source of support which people could trust and rely on compared with other social network members [9]; they provided simple relationships free from conflict [28] and they did not overstep boundaries [31]. The latter seems to be particularly beneficial for people with Autism [37] and PTSD [30].

*“The dog offers comfort in a different way to how I do, more unconditional. Åsa can hold the dog when she is feeling miserable. The dog doesn’t ask why or what’s happened”* [42].

By providing unconditional positive regard, pets promoted emotional stability through the regulation of feelings, management of stress and helping people to cope with difficult life events [27, 42]. For people living alone, pets provided a source of ‘connectedness’ [27], reassurance, and normalcy [31].

*She’s always there for me in a regular way of managing my stress. I tell her about my days, she snuggles, cuddles and sleeps with me* [34].

### Practical work - physical activity and symptom distraction

Quantitative data implicating pets in the practical work associated with mental health management pointed to the impact of dog ownership on physical activity [40] and self-reported quality of life related to physical health [29]. One study found that those with pets were more significantly likely to use ambulatory mental health care than those without [40].

Qualitative results from the studies expanded on illness specific practical work including in times of crisis [9, 28, 30–32, 35–38, 41, 42]. The main mechanism through which pets appeared to contribute to practical work was through the ability of pets to distract and disrupt attention from symptoms or upsetting experiences such as hearing voices, panic attacks or suicidal ideation [9, 27, 28, 30–32, 34, 36]. Pets contributed to practical work directly and indirectly by acting as a form of bridging tie to additional resources.

*But if I’m here and I’m having…having problems with voices and that, erm, it does help me in the sense, you know, I’m not thinking about the voices, I’m just thinking of when I hear the birds singing* [9].

One quantitative study by Stern and colleagues [30] demonstrated that whilst participants with PTSD did not report feeling less affected by painful memories or flashbacks they indicated that their pet tried to cheer them up when experiencing symptoms, indicating that the presence of their pet may have lessened a modicum of the negative impact of trauma [30].

Qualitative data pointed to the way in which pets were able to undertake the activities of practical work because of their consistent and proximate presence and through providing the opportunity for reciprocity [9, 27, 31, 34, 36]. Pets’ contribution to practical work is seemingly made possible through the provision of opportunities for routine tasks required to care for an animal, providing a positive focus for activity [9, 27, 30–32, 34, 36, 42], providing a needed challenge [36], by introducing humour into situations [9], providing a direct grounding role [30, 31, 38], lessening the negative impact of symptoms [9, 30] and reducing the stress associated with the vagaries of living with their condition [34]. One study found that participants felt that they required less medication because of this pet contribution [31].

*So the physical thing of having to brush her and take her out and feed her, check that her toenails don't need cutting, you know perhaps pick up after her if she's had an accident, things like that. Cos she can be quite demanding as you've seen, she's up and she wants attention all the time, so it… interrupts your thought process a lot of the time* [36].

*They are something that is very important in my recovery and helping me not get too depressed. Even when I was so depressed, I was kind of suicidal. I never got really bad, but I was suicidal at one time. The thing that made me stop was wondering what the rabbits would do. That was the first thing I thought of and I thought, oh yeah, I can’t leave because the rabbits need me. So they were playing a really big role in that* [32].

Pets could contribute to a sense of preparedness to take self-management action through increasing people’s positivity and self-efficacy [32, 34, 41, 42]. They encouraged their owners to stay in the present avoiding worry and ruminations about past behaviours [28, 30] or concerns about the future [34]. Pets were also considered important in terms of providing protection for their owners [28, 31]. This was particularly of value for those participants experiencing the constant vigilance associated with Post Traumatic Stress Disorder [31].

*He’ll start nudging me or hopping on me to get me into a petting session or he’ll grab my pants leg and start pulling on me or like my shirt or my arm and start pulling on me to kind of like bring me out of a flashback or anything else like that* [31].

Owners felt that their pets could sense when practical support for mental health was required and acted accordingly [9, 28, 31, 42]. However, this was not universal to all studies indicating the impact of pets cannot be fully explained by this behavioural initiation [37].

*The puppy followed Bengt’s mood from the very first day. The dog reads him inside and out, she knows exactly when to go to him and when to keep back. If Bengt is unsettled and moving around, he may stop occasionally and sit down … and then along she comes. Then she kneels down and starts to nudge and lick him, and he starts to stroke her. She also knows if he’s in conflict with us. Then she follows him … and if he hasn’t closed his door, she goes in and sits with him* [42].

*Pam named the contact itself as playing a significant role in helping her to manage anxiety attacks. She specifically described an example of when her companion dog came to her during an anxiety attack in the middle of the night: Brutus licked her face and laid next to her for the rest of the night, and contact with him immediately improved her acute symptoms* [28].

Indirectly, pets encouraged a form of behavioural activation. Pets were seen to enhance mobility [41], increase exercise [30, 35, 36, 40] and promote contact with nature [30, 36] all of which were considered beneficial to mental health.

### Pets as conduits to social interaction and emotional nourishment

A feature of the role attributed to pets in terms of mental health management in the qualitative data was the various ways in which they facilitated the quality and quantity of social interactions. Pets reportedly increased social interaction with others including friends and family [34] and with more peripheral social interactions [9, 38]. They also fostered a sense of social and community integration [9, 32, 35, 36, 38, 42]. Interestingly, one study found that dogs increased social interactions that would not have been possible without their pet (e.g. other dog walkers [36]). This was supported by some [39] but not all of the included quantitative studies [29, 32, 39, 40] indicating a complicated relationship between pets and social interaction which may be mediated by type of pet and/or number of pets [22].

*Get out of the scope of a physical disability. I mean a physical disability yeah. I can’t get through that door. I can’t get up those steps. For a mental health patient it is not the physical barrier it’s an invisible [barrier] … Yes, these guys help me interact. Butch, when we go out … when Butch and I go out, we interact because he gets so much attention and with the attention focused on him, I can get engaged in all sorts of conversations with people who like dogs, so with these guys we develop friends who are into the same thing* [38].

*That surprised me, you know, the amount of people that stop and talk to him, and that, yeah, it cheers me up with him. I haven’t got much in my life, but he’s quite good, yeah* [9].

The reasons identified in the included studies as to why pets were considered useful in terms of enhancing the amount and quality of social interactions included having the confidence to venture into new social situations with their pet, owners finding it easier to be in the presence of other people when their pet was present [30], being more open during social interactions [28] and being able to have difficult conversations with existing friends and family through their pet [34, 36].

### Biographical work - identity, a sense of self-worth and existential meaning

The data implicating pets in biographical work was mostly derived from the qualitative data. Two quantitative studies addressed this type of work; one found that despite a low effect score, pet owners performed significantly higher than non-pet owners on meaningful activity scales [39] and another found that since getting their pets individuals felt better about themselves as people [30].

Qualitative data suggested that pets provided their owners with a sense of purpose and gave meaning to their lives [41]. Often participants described how this had been diminished since diagnosis with a mental health condition but that pets helped them to overcome this and provided them with a platform for going forward with their lives [9, 38]. This sense of meaning and purpose included pets giving their owners a reason to live [9, 32], to contributing to a sense of control and empowerment [9, 31, 32, 35] and giving individuals hope for the future [9, 31]. This was considered particularly important when people were feeling consumed by illness or when self-management felt out of control [32].

*It gives me something to do, to take care of them, the cleaning of the cage, feeding them* [34].

Owners’ felt that their pets contributed directly to maintaining a consistent sense of identity and self [9, 27, 32, 36, 39, 41]. They felt pets provided a form of validation through the pride associated with successfully caring for a pet [9, 28] but also as sustaining elements of pre-illness identities including roles of mother, pet owner or animal lover [9, 36] and as being a protector of animals [28, 31].

*My best quality is that I love animals and I take care of animals… Other than that, I can’t think of anything real outstanding* [32].

Pets were also considered relevant in terms of mediating how other people viewed them [9, 42]. Pet ownership connected their owners to valued activities such as hobbies [35] and were considered a culturally sanctioned meaningful occupational and social role [38, 39]. One study also indicated that the mastery achieved through the training of animals also contributed to a positive sense of self [9].

Participants described elements of relationships with pets that were important to their mental health including the nature of relationships as simple and reciprocal, pets as understanding and honouring personal boundaries and pets not holding past behaviours against them [9, 27, 32, 36, 39, 41]. These components were often missing from other human relationship and were considered important aspects of the human/pet dyad [9].

*For Irene, taking care of her companion dog facilitated a change in her sense of self, from seeing herself as someone who “destroyed anything [she] loved” to seeing herself as a loving, nurturing protector* [28].

*There’s a lot less things to worry about. I mean you can’t…you can’t like be like if he was naughty or anything like that you’d tell him off and that was it and there’d be no hard feelings. That there’s not, you don’t get the nastiness* [9].

Pets impacted directly on the management of negative perceptions and experiences related to a diagnosis of mental illness which arose either from themselves or from others within and outside of their existing social networks [9, 28, 31, 34, 35, 39]. The mechanisms through which this appeared to operate included counterbalancing a loss of social status as a result of being diagnosed with a mental illness, providing non-judgemental acceptance often not available elsewhere [9], making owners feel wanted and valued [34, 39] and encouraging owners to feel good about themselves [28, 30]. One study proposed that companion animals symbolised abused childhood selves and that by caring for a pet they may have symbolically been caring for this part of themselves [28].

*When he comes and sits up beside you on a night, it’s different, you know, it’s just, like, he needs me as much as I need him, sort of thing* [9].

### Negative aspects of pet ownership

Despite an overall sense of the positive impact of pet ownership on the management of diagnosed mental health disorders, some negative aspects surfaced within individual quantitative and qualitative studies. This included aspects such as financial costs and housing situations, the burden of pet ownership especially if pets were unruly which could be detrimental to mental health and the guilt that owners experienced if this was not managed successfully [9, 35, 36, 38, 39]. Horses and dogs were considered the most burdensome in this regard and research highlighted the importance of matching pets to individual circumstances [36]. The early stages of pet ownership were often the most difficult for people but were concomitantly considered as an important investment in terms of future support and companionship [35]. Pets could also be seen as a barrier to aspirational goals associated with recovery such as travel [9, 35].

*When I was working it wasn't a problem, but obviously when you're on a low budget income, it does become a financial hazard, because they're just unexpected you know. That's where the issues become, do you keep them or do you…and you don't want to let them go so you're sitting there, having to cut back and scrape the bottom of the barrel to make sure they're looked after sort of thing* [36].

*I was trying to care for 3 cats of my own that I loved, stray cats in the neighbourhood I was feeding. I tried to spay the ones that appeared to be pregnant, and I was putting food out twice a day, and I was just feeling overwhelmed, just overwhelmed and more and more depressed, more a sense of failure, and finally it just got worse and worse and worse* [32].

The potential or actual loss of beloved companion animals was a major source of distress for owners [9, 32, 38, 42] but it was acknowledged that joy could still be taken in their memories once death had been come to terms with [32] and that such experiences could facilitate understanding of other difficult life events [42].

*I was very depressed by [pet’s] death. While she was getting worse, we had her home for a while and I had to make myself be strong […] It was more after her death that I kind of broke down, and just thinking about her would make me cry for a couple of weeks or more. Gradually I got to the point where I knew that it was her time. The life that she had and what she had given to me, I could always think of that. It always makes me happy* [32].

Participants described how other people including health professionals were often concerned about the safety of their pets and their ability to care for them [27, 33]. Siegel et al. demonstrated that those with HIV felt there was a perception that they should not have pets as a result of their condition [30]. This may also apply to those with mental health conditions but this was not covered in any of the included papers.

## Discussion

This review represents the first attempt to systematically identify and synthesise evidence related to the benefits of pet ownership for those with diagnosed mental health conditions. The majority of relevant data extracted for purposes of this review were qualitative and high quality prospective experimental studies were distinctly lacking. This indicates that the evidence in relation to the role of pets for the management of diagnosed mental health conditions is at an early stage and currently disparate and exploratory in nature. The use of thematic analysis informed by an existing framework led to the identification of a number of mechanisms through which companion animals were seen to support their owners to manage their mental health conditions. Very little data fell outside of the framework and what did related mostly to the demographics of pet owners. The results support the wider health benefits of companion animals for the general population [6–8]. However, the discrepancy often identified between quantitative and qualitative findings within the review and the range of factors mediating the relationship between pets and their owners identified within existing literature speaks to the complexity of this relationship. Mediating factors included the type of pet [33], the number of pets [30], perceived friendliness of pet [41] and attachment to pet [33].

Pets were implicated in emotional work because they provided a consistent and proximate source of calming support and companionship [9, 27, 30–32, 38, 39]. This was enhanced through a perception that animals could intuit when such support was needed and act accordingly providing a depth of connection that was considered particularly useful in time of crisis [9, 30, 31, 35, 36]. Companion animals contributed to practical work through their role in the distraction and disruption from upsetting symptoms and experiences [9, 27, 28, 30–32, 34, 36] through the provision of routine and a role in behavioural activation [32, 34, 41, 42]. Pets were considered important in the maintenance of a positive identity and sense of self because of the reciprocity associated with human-pet dyads [9, 27, 32, 36, 39, 41], a perception that pets accepted their owners without judgement, the sense of pride associated with successfully caring for an animal [9, 28] and supporting the management of felt and enacted stigma [9, 28, 31, 34, 35, 39]. Qualitative data demonstrated the relative strength of the role of pets in relation to all three types of work but quantitative data was unavailable to unanimously support this impact particularly in relation to practical and biographical work where quantitative evidence was distinctly lacking. Existing quantitative studies failed to include measures which adequately addressed the potential roles of companion animals as identified within the qualitative data such as self-efficacy and preparedness to take action.

Despite the mixed evidence from the quantitative data, the participants included in the review enjoyed keeping their animals and believed that they gained psychological benefit from these relationships as demonstrated by the thick descriptions derived from the qualitative data. The review demonstrated that those with diagnosable mental health problems can infer the same benefits from pet ownership as the general population and pets may have a particular role in terms of enhancing quality of life given that levels of social exclusion and stigma are likely to be greater for this population [9, 32, 35, 36, 38, 42].

Participants felt that their pets faciltiated the quality and quantity of existing social interactions and forged new relationships acting as a bridging tie to emotional nourishment [9, 32, 34–36, 38, 42]. This is likely to be of increasing importance given that social isolation is both a cause and effect of mental illness and that those with mental illness are considered one of the most socially isolated social groups [43].

Despite these identified benefits, it appeared that relationships with companion animals are not considered or incorporated into health care planning or wider health related discussions of consultations [9]. The contrary appears to occur where individuals are advised against pet ownership or experience negative attitudes from health professional in relation to their pet [33, 34]. This indicates pet ownership can create additional work for professionals in terms of managing and advising people and highlights the need for a focus on professional attitudes, which is currently lacking from the evidence base.

The findings call for cultural changes in policy towards the way in which pets can be incorporated with other support in open systems which is often left untouched or unconsidered by formal service provision. A different logic of care is required; one which values the harnessing of available and valued support identified by people, which supports individuals’ capacity to undertake valued activities (such as dog walking) and looks for support which does not engage them in unequal power relationships which can sometimes be anti-therapeutic. With increasing emphasis being placed on evidence based health care, such macro-level policy changes are likely to necessitate strengthening the underpinning evidence base given the low quality of evidence identified within the review. Further exploration of the implementation feasibility and optimal implementation models may also be required, including the potentially important role of inter-agency and third sector working.

Negative aspects of pet ownership identified in the review included concerns about potential, and the significant distress associated with the actual loss of a pet supporting previous research [44]. Evidence from those involved in natural disasters such as hurricanes suggest that pet loss can add considerably to acute trauma and increase the risk of long-term impacts [45, 46]. This highlights the potential for the loss of an animal to be of greater impact for those with diagnosable mental health conditions given the intense and positive identification reported with their pet and suggests the need to consider pets in planning and delivery of mental health care.

### Strengths and limitations

This review gains its strengths from the combination of rigorous search and extraction methods and the underlying theoretical framework which guided the analysis. To guard against bias in the undertaking of the review, two reviewers independently extracted all data and where disagreement occurred, these were discussed between authors until agreement was reached.

The level of quality across included studies was a limiting factor in this review with an average quality rating of 5.5 out of 10 for qualitative studies and 8.75 out of 10 for quantitative work (refer to Additional files 1 and 2). There was also a lack of randomised trials evaluating the impact of pet ownership on diagnosed mental health problems. This is perhaps unsurprising given the pragmatic difficulties associated with randomising individuals or families to be pet or non-pet owners within RCTs. Prospective experimental or quasi-experimental designed studies should be used to compare outcomes for pet owners and non-pet owners using measures that adequately incorporate the range of tasks in relation to each type of work as identified within this review. Given the potential benefits which might be conveyed by pets for people with mental health conditions, there is a clear need for further rigorous, high quality research, in order to consolidate these existing findings and build an evidence base on which commissioners and policy makers can base decisions.

As part of our inclusion criteria, we included only those with a diagnosable mental health problem or mental health components of a diagnosable physical health condition which may have impacted on the studies included in our review.

## Conclusion

Despite some inadequacies in the data, this review suggests that pets provide benefits to those with mental health conditions through the intensity of connectivity with their owners and the contribution they make to emotional support in times of crises together with their ability to help manage symptoms when they arise. Further rigorous research is required to test this relationship, incorporating outcomes that cover the range of roles pets may have in relation to mental health identified within this review. The research studies included in this review provide a point of debate that services and policy makers may wish to consider in the future.

## Additional files

Additional file 1: Qualitative Quality Table. Quality scores related to the included qualitative studies. (DOCX 18 kb)

Additional file 2: Quantitative Quality Table. Quality scores related to the included quantitative studies. (DOCX 18 kb)

Additional file 3: Context Table. Extracted context data from each included study. (DOCX 19 kb)

Additional file 4: Participants Table. Extracted data related to study participants from each included study. (DOCX 18 kb)

## Abbreviations

AAT: Animal assisted therapy
AIDS: Acquired immunodeficiency syndrome
DALYs: Disability adjusted life years
HIV: Human immunodeficiency virus
PRISMA: Preferred Reporting Items for Systematic Reviews and Meta-Analyses
PTSD: Posttraumatic stress disorder
UK: United Kingdom
USA: United States of America
WHO: World Health Organisation
