# Supplementary material for: The power of support from companion animals for people living with mental health problems: a systematic review and narrative synthesis of the evidence
Source: BMC Psychiatry. 2018 Feb 5;18:31. doi: 10.1186/s12888-018-1613-2 (PMC5800290; doi:10.1186/s12888-018-1613-2)
Supplement: Supplementary file 1 — Qualitative Quality Table. Quality scores related to the included qualitative studies. (DOCX 18 kb) [file 12888_2018_1613_MOESM1_ESM.docx]

| **Reference (Author, date)** | **Methodology (E)thnography (P)henomenology (G)rounded Theory (O)ther (please specify)** | **Personal characteristic of researchers reported Y/N** | **Researcher relationship to participants reported Y/N** | **Sampling method reported Y/N** | **Data collection method described Y/N** | **Data saturation discussed Y/N** | **Independent coders Y/N** | **Member checking (feedback on interpretation/coding tree) Y/N** | **Sufficient data to support findings? (Was there consistency between data presented and the findings?) Y/N** | **Contradictory data considered? (authors recognise and consider deviant cases) Y/N** | **Critical examination of researcher's own role/influence on findings? Y/N** | **Total fully met** |
| --- | --- | --- | --- | --- | --- | --- | --- | --- | --- | --- | --- | --- |
| Bradley et al., 2015 | N/S - thematic analysis | N | N | Y | Y | N | N | N | Y | N | N | 3 |
| Brooks et al., 2016 | Social network interviews | Y | Y | Y | Y | Y | Y | N | Y | Y | Y | 9 |
| Bystrom et al., 2015 | N/S - inductive thematic analysis | N | N | Y | Y | N | N | N | Y | Y | N | 4 |
| Hunt & Stein, 2007 | N/S | N | Y | Y | Y | N | Y | N | Y | N | Y | 6 |
| Pehle, Margaret A. | P | Y | N | N | Y | N | N | N | Y | N | N | 3 |
| Rijken et al., 2011 | N/A | N/A | N/A | N/A | N/A | N/A | N/A | N/A | N/A | N/A | N/A | N/A |
| Satterfield, P., 2014 | N/A | N/A | N/A | N/A | N/A | N/A | N/A | N/A | N/A | N/A | N/A | N/A |
| Stern et al., 2013 | N/A | N/A | N/A | N/A | N/A | N/A | N/A | N/A | N/A | N/A | N/A | N/A |
| Wells, 2009 | N/A | N/A | N/A | N/A | N/A | N/A | N/A | N/A | N/A | N/A | N/A | N/A |
| White, 2014 | P | Y | Y | Y | Y | Y | N | Y | Y | Y | Y | 9 |
| Wisdom, 2009 | G | N | N | Y | Y | N | Y | Y | Y | N | N | 5 |
| Zimolag & Krupa, 2009 | N/A | N/A | N/A | N/A | N/A | N/A | N/A | N/A | N/A | N/A | N/A | N/A |
| Zimolag and Krupa, 2010 | NS | N | N | Y | Y | N | N | Y | Y | N | N | 4 |
| Ford, Vicky. | GT | Y | Y | Y | Y | Y | N | Y | Y | Y | Y | 9 |
| J McNicholas. | NS | Y | N | N | Y | N | N | N | Y | N | N | 3 |
| Siegel, et al., 1999. | N/A | N/A | N/A | N/A | N/A | N/A | N/A | N/A | N/A | N/A | N/A | N/A |
| Carmack, 1991. | NS | N | N | N | N | N | N | N | N | N | N | 0 |
