# Supplementary material for: The power of support from companion animals for people living with mental health problems: a systematic review and narrative synthesis of the evidence
Source: BMC Psychiatry. 2018 Feb 5;18:31. doi: 10.1186/s12888-018-1613-2 (PMC5800290; doi:10.1186/s12888-018-1613-2)
Supplement: Supplementary file 2 — Quantitative Quality Table. Quality scores related to the included quantitative studies. (DOCX 18 kb) [file 12888_2018_1613_MOESM2_ESM.docx]

| **Reference (Author, date)** | **Methodology used (study design)** | **Description of study design provided (e.g. type of trial) Y/N** | **Inclusion/ exclusion criteria reported Y/N** | **Sampling method reported Y/N** | **Data collection method (including intervention where appropriate) described Y/N** | **Detail on study outcomes reported? Y/N** | **Detail on sample size? Y/N** | **Sufficient detail on study analysis (blinding/randomisation/ statistical methods employed) Y/N** | **Sufficient data to support interpretation of findings? Y/N** | **Drop out discussed where relevant? Y/N** | **Critical examination of study limitations? Y/N** | **Total fully met** |
| --- | --- | --- | --- | --- | --- | --- | --- | --- | --- | --- | --- | --- |
| Bradley et al., 2015 | Cross sectional survey. | Y | Y | Y | Y | Y | Y | Y | Y | Y | Y | 10 |
| Brooks et al., 2016 | N/A | N/A | N/A | N/A | N/A | N/A | N/A | N/A | N/A | N/A | N/A | N/A |
| Bystrom et al., 2015 | N/A | N/A | N/A | N/A | N/A | N/A | N/A | N/A | N/A | N/A | N/A | N/A |
| Hunt & Stein, 2007 | N/A | N/A | N/A | N/A | N/A | N/A | N/A | N/A | N/A | N/A | N/A | N/A |
| Pehle, Margaret A. | N/A | N/A | N/A | N/A | N/A | N/A | N/A | N/A | N/A | N/A | N/A | N/A |
| Rijken et al., 2011 | Household panel survey (C/S) - representative sample | Y | Y | Y | Y | Y | Y | Y | Y | Y | Y | 10 |
| Satterfield, P., 2014 | Online survey - cross sectional | Y | N | N | Y | Y | Y | Y | Y | N | Y | 7 |
| Stern et al., 2013 | Cross sectional survey. | Y | N | Y | Y | Y | Y | N | Y | Y | Y | 6 |
| Wells, 2009 | Cross sectional survey. | Y | Y | Y | Y | Y | Y | Y | Y | Y | Y | 10 |
| White, 2014 | N/A | N/A | N/A | N/A | N/A | N/A | N/A | N/A | N/A | N/A | N/A | N/A |
| Wisdom, 2009 | Survey | Y | Y | Y | Y | Y | Y | Y | Y | Y | Y | 10 |
| Zimolag & Krupa, 2009 | Survey | Y | N | N | Y | Y | Y | Y | Y | Y | Y | 7 |
| Zimolag and Krupa, 2010 | N/A | N/A | N/A | N/A | N/A | N/A | N/A | N/A | N/A | N/A | N/A | N/A |
| Ford, Vicky. | N/A | N/A | N/A | N/A | N/A | N/A | N/A | N/A | N/A | N/A | N/A | N/A |
| J McNicholas | N/A | N/A | N/A | N/A | N/A | N/A | N/A | N/A | N/A | N/A | N/A | N/A |
| Siegel, et al., 1999. | Survey and structured interview | Y | Y | Y | Y | Y | Y | Y | Y | Y | Y | 10 |
| Carmack, 1991. | N/A | N/A | N/A | N/A | N/A | N/A | N/A | N/A | N/A | N/A | N/A | N/A |
