# Supplementary material for: The power of support from companion animals for people living with mental health problems: a systematic review and narrative synthesis of the evidence
Source: BMC Psychiatry. 2018 Feb 5;18:31. doi: 10.1186/s12888-018-1613-2 (PMC5800290; doi:10.1186/s12888-018-1613-2)
Supplement: Supplementary file 3 — Context Table. Extracted context data from each included study. (DOCX 19 kb) [file 12888_2018_1613_MOESM3_ESM.docx]

| **Reference (Author, date)** | **Paper type**  J = Journal article, **C** = Conference abstract T = thesis | **Country** | **Service Setting** I = Inpatient C = Community M = mixed O = Other (please specify) NS - Not specified | **Participant Recruitment method NS - Not specified** | **Method of data collection:** Quantitative or Qualitative | **Method of data collection** I: Interview FG: Focus group O: observation C/S: C/S Survey P: Pre/post T: Trial O: Other | **Analysis type**  GT- Grounded theory, T: Thematic analysis, F: Framework analysis C: Correlational D: Descriptive statistics R: Regression O: Other |
| --- | --- | --- | --- | --- | --- | --- | --- |
| Bradley et al., 2015 | J | Australia- but study participants were world wide | C = Group of adults suffering chronic pain. | Poster advertisement in medical centres, social media, word of mouth. Supported by pain-related organisations. | Quantitative - online survey DASS-21 Depression, Anxiety and Stress Scales Qualitative - 7 semi-structured interviews | C/S I | T and D (t-tests, MANCOVA, correlation |
| Brooks et al., 2016 | J | UK | C = Those diagnosed with SMI | Invitation through invitation letters and flyers. | Qualitative - semi-structured interviews | I | F |
| Bystrom et al., 2015 | J | Sweden | C = Parents of children and adolescents diagnosed with ASD. | Convenience sampling - invitation letter attached to yearly newsletter. First 13 parents who expressed an interest were recruited. | Qualitative - focus groups. | FG | T |
| Hunt & Stein, 2007 | J | USA | O = supported housing | Invitation to all tenants living with a pet. | Qualitative - semi-structured interviews | I | NS |
| Pehle, Margaret A. | T | USA | O = Those with psychological difficulty significant enough to have sought treatment. | Adverts in Vermont newspapers, veterinarians' offices, online university newsletter and humane societies. | Qualitative - semi-structured interviews | I | O: Phenomenological |
| Rijken et al., 2011 | J | The Netherlands | C = chronic illness or disability | Household panel survey. Those participating in NPCD aged 65+ | Quantitative | CS Survey | D, R |
| Satterfield, P., 2014 | T | USA | C = chronic pain | Flyers in local treatment officers and online posting and advertisement, campus email list. Incentive offered. | Quantitative | Online Survey | D and R |
| Stern et al., 2013 | J | USA | C = PTSD diagnosis | Clinician invitation. | Quantitative | Survey | D |
| Wells, 2009 | J | Northern Ireland | O = Chronic fatigue syndrome | Open invitation on CFS website and newsletters | Quantitative and Qualitative content analysis | Survey | D and R and O |
| White, 2014 | T | USA | C = PTSD diagnosis | Flyers distributed via community organisations and they advertised the study through social media. | Qualitative - semi-structured interviews | I | O and T: Phenomenological |
| Wisdom, 2009 | J | USA | C = diagnosis of schizophrenia, schizoaffective disorder, or affective psychosis. | Letter invitation, identified through health records. | Mixed methods (quant survey and qualitative interviews) | I and C/S | D and GT |
| Zimolag & Krupa, 2009 | J | Canada | C = | Invitation pack to professionals. | Quantitative | Survey (professional completed and service user completed) | D |
| Zimolag and Krupa, 2010 | J | Canada | C = | Participant invited from existing database. | Qualitative - case study | I | T |
| Ford, Vicky. | T | UK | C: Mental Health charities. | Participants attending community-based support groups were invited to take part in this research, through direct contact with group facilitators. | Qualitative | I | GT |
| J McNicholas. | T | UK | C - Children and young people diagnosed with ASD. | Invited through advert in newsletter for carers of people with autism. | Qualitative | I | NS |
| Siegel, et al., 1999. | J | USA | C: Patients with presence of HIV infection |  | Quantitative | S and structured interview | D and R |
| Carmack, 1991. | J | USA | C: Patients with HIV/AIDS | NS | Qualitative | I | NS |
