# Supplementary material for: The power of support from companion animals for people living with mental health problems: a systematic review and narrative synthesis of the evidence
Source: BMC Psychiatry. 2018 Feb 5;18:31. doi: 10.1186/s12888-018-1613-2 (PMC5800290; doi:10.1186/s12888-018-1613-2)
Supplement: Supplementary file 4 — Participants Table. Extracted data related to study participants from each included study. (DOCX 18 kb) [file 12888_2018_1613_MOESM4_ESM.docx]

| **Reference (Author, date)** | **Number of pet owners** | **Number PO approached O- Open advertisement (e.g. poster) NS - not specified** | **PO Number female pet owners NS- Not specified** | **PO diagnosis (if applicable) -S (SMI) : Sample people with SMI -M (Mixed): -A/D Anxiety/depression G: general MH O: Other** | **PO mean age NS- Not specified** | **PO age range NS- Not specified** | **PO ethnicity NS- Not specified** |
| --- | --- | --- | --- | --- | --- | --- | --- |
| Bradley et al., 2015 | 173 participants (132 companion-animal owners, 39 non owners and 2 not specified). 7 qualitative participants. | O - numbers not specified | NS Quantitative 7 qualitative | Other - study looked at those with self-reported pain conditions (fibromyalgia, nerve damage, arthritis, disc problems and headaches or migraines). | 47.06 | 19-72 | NS |
| Brooks et al., 2016 | 25 | O - numbers not specified | 17 68% | SMI (Bi-polar or schizophrenia) | NS | NS | 100% White. |
| Bystrom et al., 2015 | 13 parents of children with AHD and companion animal. | NS | NS | NS | NS - only information on the children and not the participants. | NS - only information on the children and not the participants. | NS |
| Hunt & Stein, 2007 | 44 | 46 | NS | SMI | NS | NS | NS |
| Pehle, Margaret A. | 8 | NS | 8 | M | NS | 25-67 | 100% white. |
| Rijken et al., 2011 | 281 | NS | NS | O: Chronic illness or disability | NS | NS | NS |
| Satterfield, P., 2014 | 31 | NS | NS) | O: Chronic pain | NS | NS | NS |
| Stern et al., 2013 | 30 | NS | 3 | O: PTSD | 56.9 | 34-67 | Hispanic 16, non-Hispanic white 9, African-American 1, American Indian 1, Multiracial 3. |
| Wells, 2009 | 98 | O - not specified | NS | O: Chronic fatigue syndrome | NS | NS | NS |
| White, 2014 | 12 | O | 2 | O: PTSD | NS | NS | NS |
| Wisdom, 2009 | 101 | 418 | 50 | SMI: schizophrenia, schizoaffective disorder, bipolar disorder, or affective psychosis | 47.3 | NS | NS |
| Zimolag & Krupa, 2009 | 20 | 36 | 13 | Mood disorder, schizophrenia. | NS | NS | NS |
| Zimolag and Krupa, 2010 | 1 | 1 | 100% | Bipolar | Mid-50s | N/A | NS |
| Ford, Vicky. | 10 | NS | 6 | Severe and enduring mental health problems. | NS only age ranges given. | NS | NS |
| J McNicholas. | 3 | Open | 0 (men only) | Autism | 15 | 11-22 years | NS |
| Siegel, et al., 1999. | 907 | 2187 | 0 (men only) | O: HIV | 38 years | NS | White, non Hispanic 1794 (96%), other 78 (4%). |
| Carmack, 1991. | 11 | NS | 0 (men only) | O: HIV/Aids | NS | NS | NS |
